# Method for Bottle Opening with a Dual-Arm Robot

**DOI:** 10.3390/biomimetics9090577

**Published:** 2024-09-23

**Authors:** Francisco J. Naranjo-Campos, Juan G. Victores, Carlos Balaguer

**Affiliations:** RoboticsLab, Systems and Automation Engineering Department, University Carlos III of Madrid, 28911 Leganés, Spain; jcgvicto@ing.uc3m.es (J.G.V.); balaguer@ing.uc3m.es (C.B.)

**Keywords:** assistive technology and rehabilitation engineering, autonomous robotic systems, robot manipulators, perception and sensing, machine learning, reinforcement learning control, position and force control

## Abstract

This paper introduces a novel approach to robotic assistance in bottle opening using the dual-arm robot TIAGo++. The solution enhances accessibility by addressing the needs of individuals with injuries or disabilities who may require help with common manipulation tasks. The aim of this paper is to propose a method involving vision, manipulation, and learning techniques to effectively address the task of bottle opening. The process begins with the acquisition of bottle and cap positions using an RGB-D camera and computer vision. Subsequently, the robot picks the bottle with one gripper and grips the cap with the other, each by planning safe trajectories. Then, the opening procedure is executed via a position and force control scheme that ensures both grippers follow the unscrewing path defined by the cap thread. Within the control loop, force sensor information is employed to control the vertical axis movements, while gripper rotation control is achieved through a Deep Reinforcement Learning (DRL) algorithm trained to determine the optimal angle increments for rotation. The results demonstrate the successful training of the learning agent. The experiments confirm the effectiveness of the proposed method in bottle opening with the TIAGo++ robot, showcasing the practical viability of the approach.

## 1. Introduction

The integration of robotics into home environments is experiencing a surge in demand, driven by recent technological advancements. In these settings, robots can be designed to fulfill a variety of human needs, a specialized area known as Assistive Robotics (AR). This field is defined as the use of robots to directly assist and interact with humans in an adaptive manner [1]. For example, utilizing robots to provide companionship and entertainment for hospitalized or elderly individuals has spurred innovations in social human–robot interaction [2,3,4,5].

**Figure 1:**
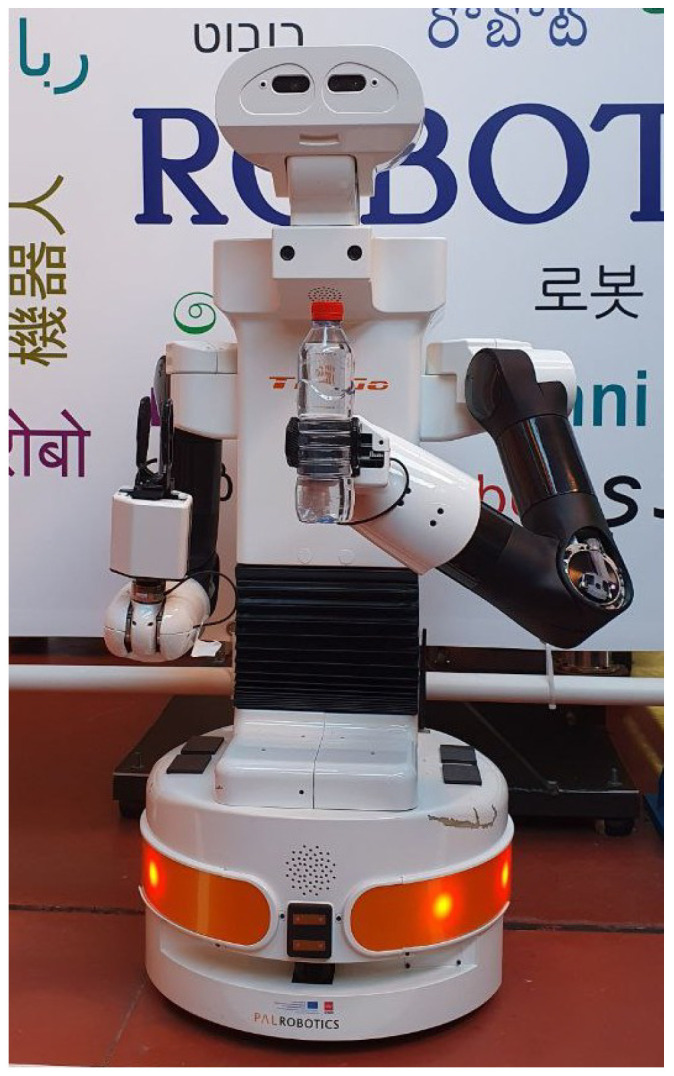
TIAGo++ dual-arm mobile manipulator robot with a bottle in one gripper.

Furthermore, an essential aspect of assistive robotics is its focus on aiding individuals with mobility issues who require help with routine daily tasks. This includes the growing number of elderly individuals and those with spinal cord injuries, who often need such assistance. Depending on the degree of injury, the level of limitation may vary [6,7]. For some, one particularly challenging task for these individuals is opening bottles. This research aims to address this specific need by implementing a manipulation task using the TIAGo++ robot, which is presented in Figure 1. By focusing on this task, the work highlights the practical applications of robotics in enhancing the independence and quality of life for individuals with mobility impairments.

Various robotic approaches have been developed to assist individuals with mobility issues. These approaches include manipulator robots designed to help users with manipulation tasks [8,9,10,11]. Additionally, mobile robots have been applied to serve humans as autonomous transport devices or walking guides in their environments [12,13,14,15,16]. There are also mixed applications involving mobile manipulator robots that can perform both object manipulation and delivery tasks [17,18,19]. Moreover, dual-arm robots are of particular interest in this field because they offer greater possibilities for manipulation tasks.

Interest in dual-arm manipulation is growing due to the increasing demand for using robots in environments designed for humans. In such environments, wheeled and legged humanoid robots are especially well-suited because their design resembles that of a human. There is a substantial body of knowledge on robotic manipulation [20,21,22] and, specifically, on dual-arm manipulation [23]. Research in this area includes various advanced control methods such as input-output linearization methods, nonlinear feedback techniques, hybrid force/position control, impedance control, sliding mode control, robust adaptive control, neuro-adaptive control, and even simple control based on proportional-derivative (PD) control with gravity compensation.

On the other hand, the task of unscrewing a bottle has been addressed by the robotics field from various perspectives, including the use of dual-arm manipulation. Control theory often suggests defining a precise trajectory and ensuring that the robotic arm follows it accurately. In this approach, the accurate control of the contact between the end-effector and the bottle cap is crucial to correct any slippage. This has been successfully achieved through position and force control [24] or by employing complex force sensors [25]. Another prevalent approach to the robotic bottle unscrewing task involves human demonstration using a sensorized glove [26,27,28]. The signals recorded during the demonstration are mapped to the actuators of the end-effector to reproduce similar movements. However, these implementations often lack adaptability and are not well-integrated into a comprehensive task pipeline.

To increase the adaptability of the robot, this paper proposes the use of Deep Reinforcement Learning (DRL) methods to accomplish the bottle unscrewing task. In classic Reinforcement Learning (RL), the problem is considered as a Markov Decision Process (MDP), defined as a tuple $\left(S,A,p,\rho_{0},r\right)$, where *S* is the state space, *A* is the action space, p(s,a) is the dynamics function, $\rho_{0}$ is the initial state distribution, and *r* is the reward function [29]. The objective of this framework is to obtain a policy π(a|s) that maximizes the expected sum of rewards. DRL extends this approach by using deep artificial neural networks to approximate the necessary functions [30]. This addition allows for the representation of more complex functions and the handling of high-dimensional state and action spaces, significantly enhancing the agent’s ability to learn intricate and abstract patterns within the data.

Different approaches exist to solve the dual-arm manipulation problem with DRL methods. In alignment with control theory, DRL has been applied to develop stochastic policies for force control of the end-effector, a technique known as consensus-based control [31]. Other researchers have utilized DRL to control motion by considering only one kinematic chain [32]. Conversely, another perspective involves applying Multi-Agent Deep Reinforcement Learning (MADRL), where each robotic arm is treated as an individual agent, and coordination strategies between them are implemented [33,34]. This approach allows for more dynamic and flexible interactions between the arms, enhancing the overall manipulation capability.

To address the specific task of opening a bottle, this paper proposes a detailed process pipeline. Initially, the positions of the bottle and cap are obtained through computer vision techniques. Following this, a classical pick-and-place approach is employed, where the robotic arm performs trajectory planning to pick up the bottle and grip the cap. The unscrewing motion is then executed using a combination of position and force control schemes, ensuring that both grippers follow the predefined path accurately. Within the control loop, the vertical axis is managed through measurements from force sensors, while the rotational motion is controlled using DRL methods [30]. This integrated approach leverages the precision of traditional control methods and the adaptability of DRL to effectively perform the bottle-opening task, demonstrating a significant advancement in dual-arm robotic manipulation.

## 2. Materials and Methods

This section provides a comprehensive overview of the materials and methodology employed to accomplish the bottle-opening task using the TIAGo++ robot. It is organized into three subsections: TIAGo++, the definition of the task and the implemented method to execute the task.

### 2.1. Tiago++

The robot used in this work is the research-oriented TIAGo++ from PAL Robotics (See https://blog.pal-robotics.com/tiago-bi-manual-robot-research/, accessed on 12 July 2024), which is shown in Figure 1. TIAGo++ features a mobile base with differential wheels, ensuring smooth and stable navigation. Combined with a laser sensor, it can map the environment and implement autonomous navigation effectively.

For manipulation tasks, TIAGo++ is equipped with two robotics arms with seven degrees of freedom each one, a prismatic torso, and grippers as end-effectors. The grippers are designed with a rubberized surface to enhance grip and minimize slippage when handling objects. This feature is particularly useful for tasks such as picking, placing, and manipulating items, ensuring a firm and secure hold. Additionally, the robot includes an RGB-D camera mounted on a pan-and-tilt system located at the head position, which provides comprehensive and effective perception of the environment. This combination of advanced grippers and perception capabilities makes the TIAGo++ robot well-suited for a wide range of complex manipulation tasks.

The robot is entirely based on the ROS (Robot Operating System) Noetic (See https://www.ros.org/, accessed on 23 July 2024) framework, providing a flexible and widely-used platform for robotic development. This makes TIAGo++ an ideal platform for robotics research, allowing for experimentation with navigation, manipulation, human–robot interaction and artificial intelligence.

### 2.2. Definition of the Bottle Opening Task

**Figure 2:**
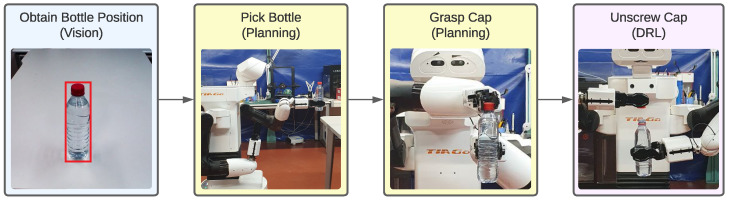
Process pipeline to achieve the opening of the bottle with the dual-arm robot.

The definition of the bottle-opening task serves as a comprehensive framework for understanding the intricate processes and methodologies required for its successful execution. This task is structured into a sequence of well-defined sub-tasks: detecting the bottle, picking it up, grasping the cap, and ultimately unscrewing the cap. The overall process pipeline is illustrated in Figure 2, which provides a visual representation of the steps involved.

Initially, to effectively manipulate the bottle, it is imperative to accurately locate the elements to be handled within the workspace. This begins with determining the precise position of the bottle, which necessitates advanced perception capabilities. Sophisticated computer vision systems are employed to identify the bottle within its environment. Once the bottle is detected, the next step involves distinguishing the cap from the body of the bottle. This requires analyzing the bottle’s shape and structure to accurately pinpoint the cap’s location, ensuring that subsequent actions are precise and effective.

Regarding the bottle, specific constraints are set to clearly define the task. The bottle to be manipulated is assumed to be of a standard small size, in accordance with the guidelines established by the International Society of Beverage Technology (ISBT) for plastic bottle fabrication [35]. Additionally, it is assumed that the cap and the body of the bottle exhibit a significant color contrast, which aids in distinguishing between the two components. Finally, the cap is considered to be normally closed, meaning the thread is not excessively tightened and can be opened with a manageable amount of force.

Following the identification phase, the task progresses to the actual manipulation process. The robotic arms must be guided to securely pick up the bottle and firmly grasp the cap. This involves implementing trajectory planning algorithms for both robotic arms to ensure they reach and maintain the desired poses accurately.

**Figure 3:**
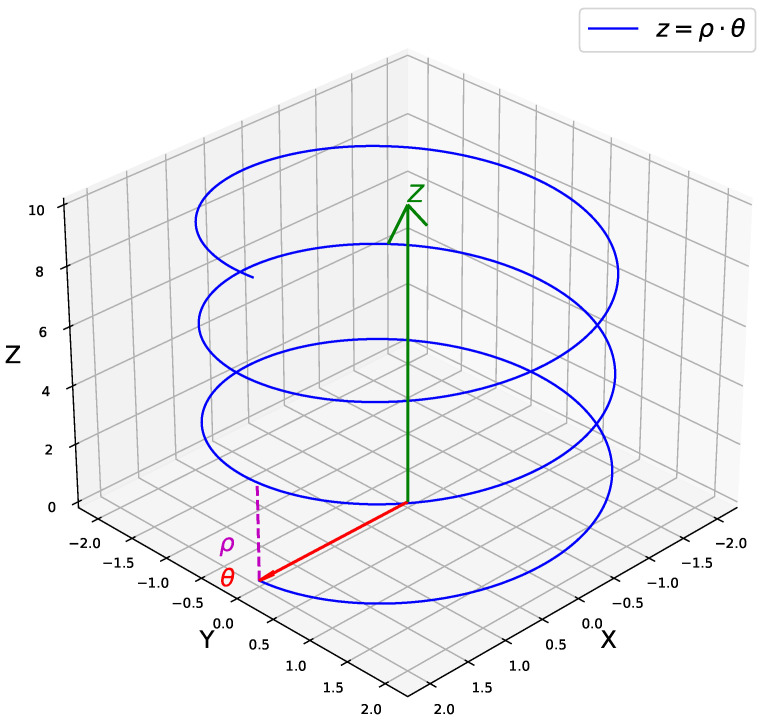
A 3D representation of a helix curve shown in blue. The radial coordinate θ is highlighted in red, while the vertical coordinate *Z* is shown in green. The pitch of the helix (ρ) is depicted with a magenta dashed line.

Once in position, the unscrewing process can be initiated. This step involves making the cap move along the thread of the bottle. The thread follows a helical path, which can be described by the Equation (1) and is depicted in Figure 3.

(1) z=pθ

where *z* represents the vertical linear displacement, θ denotes the angle of rotation, and *p* is a constant that defines the pitch of the helix. To unscrew the cap effectively, it must be moved along the vertical Z-axis while simultaneously rotating around the same axis relative to the bottle. This dual-motion ensures that the cap follows the helical path of the thread, facilitating its removal.

**Figure 4:**
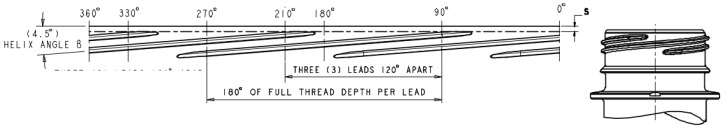
Thread standard for bottles with a 26 mm aperture. On the left, the thread development from the outside is shown, and on the right, the profile view is provided. Adapted from [35].

Additionally, the ISBT standard guidelines define the specific thread pattern for bottle caps. These guidelines include the thread’s pitch and extension, as shown in Figure 4. According to this standard, the stabilized angle of the thread that must be covered is $\delta \theta =\theta_{open}-\theta_{close}=\pi$. Thus, rotating the cap by this angle, combined with the appropriate vertical displacement, will result in successful unscrewing. This principle is fundamental to implementing the final unscrewing movement in the process pipeline.

In summary, the bottle-opening task is a multifaceted process involving precise detection and manipulation. It encompasses detecting the bottle, securely picking it up, accurately grasping the cap, and finally unscrewing it. Each step in this sequence is critical for the successful completion of the task, and the implementation details of this process are elaborated in the following sections.

### 2.3. Method for Bottle Opening

The implementation of the proposed method for bottle opening is described below. This implementation is divided into the following stages: detection of the bottle and cap, the process of picking up the bottle and grasping the cap, and finally, the unscrewing process.

#### 2.3.1. Determining Bottle and Cap Positions

Initially, the robot is positioned in front of a table where the bottle is placed. The methodology begins with this initial state and involves using an RGB-D camera and computer vision to determine the Cartesian coordinates of the bottle and the cap. This procedure relies on the premise that there is a color contrast between the bottle’s body and its cap.

**Figure 5:**
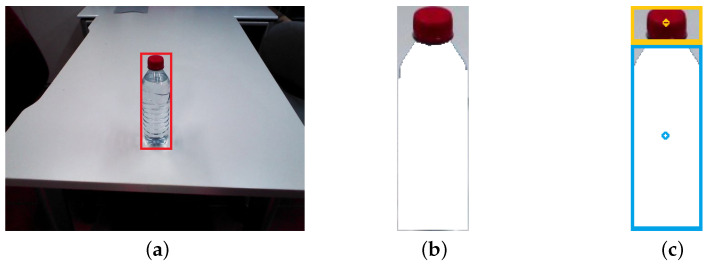
Image processing to obtain centroids of bottle cap and body. (**a**) ROI of the bottle detected with YOLO, marked in red. (**b**) Watershed applied to obtain a mask of the bottle’s body, shaded in white. (**c**) Centroids of body and cap of the bottle, marked in blue and yellow.

Figure 5 depicts the RGB image processing pipeline. The image is initially input to a pre-trained instance of YOLOv8 [36], a convolutional neural network-based detector that identifies various entities within an image. YOLOv8 provides the bounding box of the bottle (Figure 5a), thereby defining the region of interest (ROI) for subsequent processing.

Subsequently, the watershed transform [37], an image segmentation algorithm, is applied. This algorithm is instrumental in separating contiguous yet distinct objects in an image. The Watershed algorithm is initiated from the centroid of the ROI and generates a mask representing the bottle’s body (Figure 5b). Subsequently, the ROI is subdivided into two bounding boxes, as depicted in Figure 5c: one encompasses the generated mask, which represents the bottle’s body, and the other comprises the remainder of the ROI, which represents the cap. Then, the centroid of both bounding boxes are computed.

Finally, leveraging the intrinsic parameters and depth information obtained from the RGB-D camera, the centroid pixels are converted into 3D Cartesian coordinates relative to the base frame of the robot.

#### 2.3.2. Bottle Picking and Cap Grasping

After determining the bottle’s position relative to the robot, the picking action is executed using the left arm. This process builds upon previous implementations of manipulation tasks performed with a TIAGo robot [11]. Specifically, the path planner Single-query Bi-directional Lazy collision checking (SBL) [38] is employed to generate safe trajectories for the arm, guiding it through designated end-effector poses.

**Figure 6:**
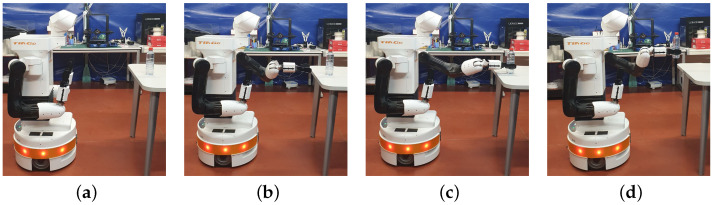
Frames of the sequence of picking the bottle. (**a**) Initial state after vision. (**b**) Arm in pre-pick configuration. (**c**) Grasp the bottle. (**d**) Retreat the arm.

The bottle-picking operation is a straightforward process, approaching the bottle with the end-effector and grasping it. This sequence of poses is illustrated in the frames shown in Figure 6.

**Figure 7:**
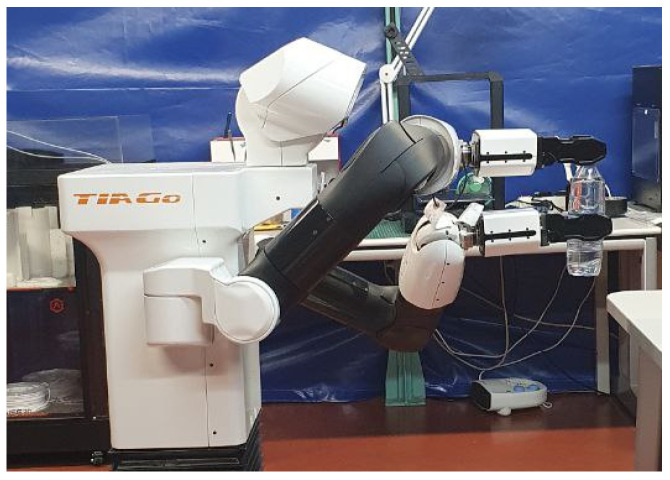
Robot grasping the bottle and the cap.

Once the bottle pick has been completed, the right arm proceeds to grasp the cap. The position of the cap is predefined based on the known translation of the bottle, which is determined from its detected position to its position after being picked up. Once more, the path planner is employed to direct the gripper toward the cap. Subsequently, the right gripper closes until grasping. The robot remains as depicted in Figure 7.

#### 2.3.3. Unscrewing the Bottle Cap

To execute the bottle cap unscrewing movement, a position and force control scheme is implemented to follow a screwing path based on the thread of the bottle cap, as previously defined as a helix path. Starting from the remained robot pose shown in Figure 7 with a relative angle between the grippers $\delta \theta_{0}=0$, the path consists of rotating both grippers around the vertical axis *Z* to achieve a relative angle $\delta \theta_{goal}=\pi$, accompanied by linear movement along the same axis *Z*, while the other coordinates remain constrained.

**Figure 8:**
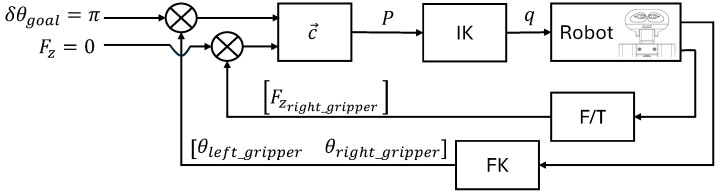
Control scheme for force and position. The controller, represented by the control vector $\vec{c}$, provides the desired poses *P* of both grippers. These poses are transformed into joint values *q* using inverse kinematics (IK). The achieved poses of the robot are determined via forward kinematics to obtain the angles θ along the *Z* axis for positional feedback. Additionally, the force along the *Z* axis of the right gripper is measured by the force/torque sensor (F/T) for force feedback.

The control scheme manipulates the poses of both end-effectors, focusing exclusively on the angular and linear components along the *Z* axis. At each time-step during unscrew execution, a control vector ${\vec{c}}^{\;t}$ determines new desired end-effectors poses to follow the specified unscrew goal, which is commanded to the arm joints via inverse kinematics (IK). This vector computes the desired new poses using the current robot pose obtained by forward kinematics (FK) and the force read by the force/torque sensor. In Figure 8 we present this control scheme.

Angular control is facilitated through Deep Reinforcement Learning (DRL) integrated into the feedback loop, ensuring precise angle adjustments. Meanwhile, control over the linear *Z* axis involves gravity compensation aimed at achieving zero force, thereby enabling unrestricted movement. Thus, the control vector ${\vec{c}}^{\;t}$ is formally defined in Equation (2)

(2) $${\vec{c}}^{\;t}=\left\{\begin{array}{l} {\vec{c}}_{\theta_{right\_gripper}}^{\;t}=\theta_{right\_gripper}^{t}+\Delta \theta_{right\_gripper}\\ {\vec{c}}_{\theta_{left\_gripper}}^{\;t}=\theta_{left\_gripper}^{t}+\Delta \theta_{left\_gripper}\\ {\vec{c}}_{z_{right\_gripper}}^{\;t}=z^{t_{right\_gripper}}+\left(K_{f}F_{z_{right\_gripper}}^{t}-K_{b}\right) \end{array}\right.$$

where *t* is the current time-step, $\left({\vec{c}}_{\theta_{right\_gripper}}^{\;t},{\vec{c}}_{\theta_{left\_gripper}}^{\;t},{\vec{c}}_{z}^{\;t}\right)$ are the angular and linear components in *Z* of ${\vec{c}}^{\;t}$, $\left(\theta_{right\_gripper}^{t},\theta_{left\_gripper}^{t}\right)$ are the position of the angle in *Z* of both grippers at time-step *t*, $\left(\Delta \theta_{right\_gripper},\Delta \theta_{left\_gripper}\right)$ are the angle increment given by the DRL agent for each gripper, $z_{right\_gripper}$ is the position in *Z* of the upper right gripper, $K_{f}$ is an empirical-tuned proportional gain of the force control, $F_{z}$ is the force’s vertical component measured in the right gripper, and $K_{b}$ is a bias force compensation. In this way, the vector ${\vec{c}}^{\;t}$ provides the desired position for the next time-step which is then translated to the joint space using inverse kinematics. The following describes the gravity compensation and the implementation of the DRL agent in detail.

##### Gravity Compensation

Effective gravity compensation in TIAGo’s arm is based on the readings from the force sensor located at the wrist. This process requires the estimation of two constants that appear in Equation (2): $K_{f}$ and $K_{b}$.

The constant $K_{b}$ is used to compensate for the bias in the force sensor. To determine $K_{b}$, the force exerted by the end end-effector exposed just to gravity was measured. The average of these measurements was taken as $K_{b}$.

The constant $K_{f}$ defines the sensitivity of the gravity compensation. It has been experimentally approximated to ensure that the resistance encountered by the end end-effector when unscrewing is effectively counteracted.

##### DRL Agent

The DRL agent was implemented using the Gymnasium library (see https://gymnasium.farama.org/index.html, accessed on 27 August 2024). Its goal is to provide angle increments for both grippers, targeting the desired relative angle of $\delta \theta_{goal}=\pi$. The environment defines the pose of both grippers and is characterized by the state space, action space, and reward function:

- State space S is represented by a tensor for covering the matrix rotation and position vector of both grippers as defined in expression (3):
  (3) $$\begin{array}{c} S:=\left\{\begin{array}{cc} \left[R_{\mathrm{left}}\;p_{\mathrm{left}}\right]\;; & \left[R_{\mathrm{right}}\;p_{\mathrm{right}}\right] \end{array}\right\}\\ S\in R^{3\times 4\times 2},\;R_{\mathrm{left}},\;R_{\mathrm{right}}\in R^{3\times 3},\;p_{\mathrm{left}},\;p_{\mathrm{right}}\in R^{3\times 1} \end{array}$$
- Action space A is represented by a vector of the angle increment in the *Z* axis for both grippers as defined in the expression (4):
  (4) $$\begin{array}{c} A:=\left\{\begin{array}{cc} \Delta \theta_{left} & \Delta \theta_{right} \end{array}\right\}\\ A\in R^{2},\;\Delta \theta_{left},\;\Delta \theta_{right}\in R \end{array}$$
- The reward function is defined as the negative difference between goal $\delta \theta_{goal}$ and current $\delta \theta_{t}$ as defined by the Equation (5):
  (5) $$r\left(s_{t}\right)=-\left|\delta \theta_{goal}-\delta \theta_{t}\right|=-\left|\delta \theta_{goal}-\left(\left|\theta_{left\;t}-\theta_{right\;t}\right|\right)\right|,$$
  where *t* is the time-step and $\theta_{left\;t},\theta_{right\;t}$ are the angle in the *Z* axis of both grippers computed from their rotation matrix.

The interaction between the environment and the robot relies on inverse kinematics (IK) and forward kinematics (FK). In each state, the agent predicts an action, which consists of angular increments. These increments are translated into rotation matrices and applied to the current state of the robot to obtain new poses. Through IK, the new poses are converted into joint values for both arms. Before issuing commands to the controllers, potential singularities in the resulting poses are carefully examined to avoid unsafe configurations. After movement is executed, FK is used to determine the achieved new poses, and therefore, the new state of the environment.

## 3. Results

This section presents the experimental results. Initially, the outcomes of training the DRL agent in a simulated environment are presented. Following this, the results from experiments involving the complete execution of the process are described.

### 3.1. DRL Agent Training

**Figure 9:**
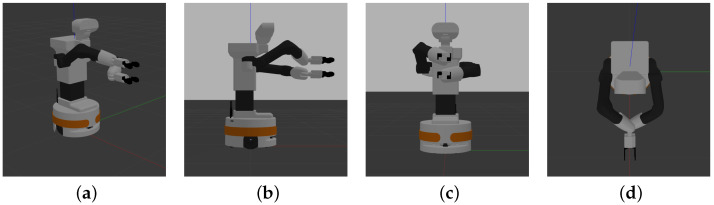
Simulation of the TIAGo++ robot in the initial position for unscrewing a bottle. (**a**) Perspective. (**b**) Profile. (**c**) Elevation. (**d**) Plan.

The DRL agent has been trained in simulation. In the simulation, the robot’s arms are initially positioned as if the bottle were being picked, as shown in Figure 9, but the bottle model is not included. This allows the agent to learn the increments needed to achieve the rotation without considering the effects of gravity compensation.

**Figure 10:**
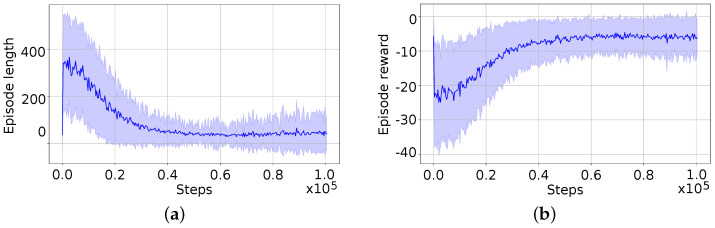
Training summary values of DRL agent over 100 runs. (**a**) Episode length, mean and standard deviation across 100 runs. (**b**) Episode reward, mean and standard deviation across 100 runs.

For the training, the Proximal Policy Optimization (PPO) algorithm [39] was employed using the stable-baselines3 library (see https://stable-baselines3.readthedocs.io/en/master/modules/ppo.html, accessed on 27 August 2024), with a learning rate of α=0.001, a discount factor of γ=0.99, and initialization of each training episode in poses as shown in Figure 9. The training was conducted over 100 runs of 100,000 steps. Figure 10 presents graphs of two summary metrics: the mean episode reward over steps and the mean episode length over steps. In Figure 10a, the observed progressive decrement of the episode length indicates that the agent optimizes the task execution until it converges to the minimum needed steps. On the other hand, in Figure 10b the increment of the mean reward can be seen until it converges to the maximum value, which matches with the convergence of the episode length.

This trained policy has been then applied to the real robot and its performance is presented in the following experiment.

### 3.2. Complete Execution of the Process

This experiment was conducted to verify the correct execution of the implemented method. The initial setup, as previously described, involved the robot positioned in front of the table where the bottle is placed. The sequence of processes was successfully executed with a 100% success rate, with the robot consistently achieving the goal of opening bottles within an execution time of 60.4±2.9 seconds as shown in the Table 1.

**Figure 11:**
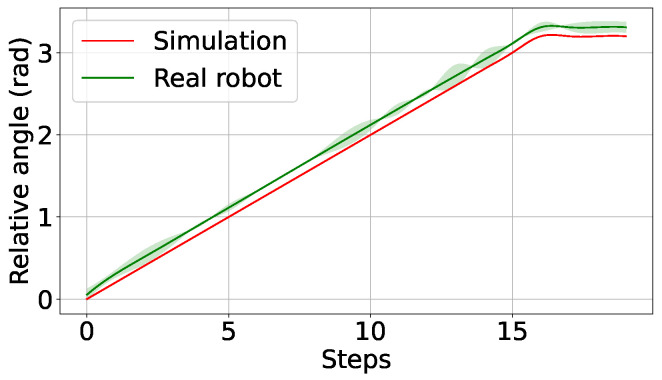
Relative angle over steps in simulation and real robot, mean and standard deviation over 10 executions.

Furthermore, the control system designed to manage both force and position has demonstrated excellent efficacy in achieving the desired outcomes, even with rotation controlled by an agent trained in simulation. The transfer of the system has proven to be accurate, as illustrated in the graph in Figure 11. The graph presents the relative angle between the grippers in simulation and the real robot, along with the associated error.

## 4. Discussion

The experimental results presented in this study highlight the efficacy of the implemented method for the bottle-opening task using the TIAGo++ robot. In view of the results, several aspects can be discussed regarding the training and transfer of the agent and the completion of the entire task with the task constraints. Additionally, a comparison between our approach and the current state of the art is discussed.

### 4.1. Agent Training and Transfer

The agent was trained in simulation under conditions different from reality but similar. The main difference is the absence of the bottle model in the simulation. This means it was trained under conditions where the poses do not receive position increments on the vertical Z-axis due to gravity compensation and the resistance of the cap following the bottle’s thread.

However, when employing the agent in the real situation with the complete control system and the bottle, it behaves appropriately with actions (angle increments) close to those given in the simulation as depicted in the graph of Figure 11. This is mainly because the position increments on the Z-axis introduced by gravity compensation are extremely small and smooth, following the thread.

Nevertheless, the use of DRL techniques has greater potential for achieving control of the bottle’s unscrewing. For future work, it would be interesting for the agent to learn which actions to take for each component of the pose of grippers.

### 4.2. Execution and Constrains of the Task

The complete task execution demonstrated strong performance with a 100% success rate. This was primarily due to the stringent constraints established, which ensured robust implementation as long as specific conditions were met: a small, standard-sized bottle, a color difference between the cap and the rest of the bottle, the cap being not tightened and the robot positioned in front of the table where the bottle is located.

These constrain this implementation, surging some limitations and areas for future improvement. One negative aspect is the dependency on the color difference between the cap and the bottle. If the cap and bottle are similar in color, the detection of the cap can not be accurately conducted and subsequent manipulation will fail.

For future work, improvements could include developing more sophisticated detection algorithms that do not rely solely on color differences. Enhancing the robustness of the system to handle a wider variety of bottle sizes and shapes would also be beneficial.

Regarding the cap’s tightness, there have been previous approaches that involve using sophisticated slip detection sensors to monitor slippage and integrate this information into the control loop. However, in this implementation, the need for such sensors has been deemed unnecessary due to the cap not being overly tightened and the use of compliant grippers. The results have demonstrated that slip detection can be avoided under these conditions. Nevertheless, incorporating slip detection could become relevant if the implementation is extended to handle a wider range of bottle states and conditions in the future.

### 4.3. Comparison with the State of the Art

As mentioned in the introduction, the screw process using robotic arms has already been approached from different perspectives. The three main approaches are generating trajectories with force-torque control [24], utilizing complex force-torque sensors to measure slippage [25], and using human demonstration [28]. Table 2 shows the unscrewing execution times achieved with these approaches compared to ours. Only the unscrewing task is compared, as these other works primarily focus on this specific process.

Our approach achieves the shortest execution time, not only because of the adaptability provided by the learning algorithm, which works alongside force control in an embodied control system, but also because the control system is well-matched with the design constraints of the bottle.

In contrast, other approaches primarily focus on sensing the force, torque, or slippage applied by the end-effector to the cap. Our system indirectly measures the friction caused by the thread during the unscrewing process and adapts accordingly. This makes our method more straightforward and, as a result, faster.

### 4.4. Future Works

Future research could focus on expanding the DRL agent’s capabilities by incorporating force control, enabling the system to apply precise amounts of force during the bottle opening process. This would allow the robot to handle more delicate tasks, such as working with fragile bottles or tightly sealed caps.

Moreover, integrating comprehensive visual information into the observation space, including compressed images and depth data, would enable the agent to learn how to act based not only on force control but also on visual cues from its environment.

Enhancing the learning algorithm to better manage the gripper’s pose could result in more accurate and stable gripping, reducing the risk of slippage or misalignment during the unscrewing process. This improvement would also make the system more adaptable to a wider range of bottle designs, materials, and environmental conditions, such as varying levels of friction or the presence of obstacles.

## 5. Conclusions

This paper presents an advanced methodology for robotic bottle opening using the dual-arm TIAGo++ robot, which integrates vision, manipulation, and learning techniques to perform the task effectively. The approach utilizes an RGB-D camera to capture the positions of both the bottle and the cap. Following this, the robot plans and executes precise trajectories to pick up the bottle and grip the cap. The unscrewing process is managed through a combination of position and force control schemes, ensuring accurate adherence to the designated path by the grippers. The control loop incorporates force sensor data for adjustments along the vertical axis and employs a Deep Reinforcement Learning (DRL) algorithm to manage rotational movement.

The experimental results validate the effectiveness of this approach, demonstrating that a process pipeline incorporating various techniques can be integrated to achieve the bottle-opening task. Notably, the learning agent, which was successfully trained in simulation, effectively transferred to real-world scenarios. This successful transfer can be attributed to the relatively simple nature of the task. The integration of DRL has proven to be a significant asset, enabling the robot to handle complex manipulation tasks by seamlessly combining force and position control.

Despite these successes, the implementation is subject to certain constraints in task definition. These constraints have simplified the implementation by, for example, allowing the disregard of complex issues such as slippage and cap identification based on color variation. However, they also limit the adaptability of the system to different scenarios.

Future research could also explore extending the capabilities of the DRL agent to encompass force control and integrate comprehensive visual information for improved bottle and cap detection. Enhancing the learning algorithm to better manage the gripper’s pose and adapt to diverse task conditions will further advance the effectiveness and versatility of robotic assistance in bottle opening tasks.

In summary, this paper presents a significant step forward in robotic manipulation for bottle opening, showcasing the potential of combining vision, manipulation, and learning techniques. Continued development and refinement of the system will enhance its applicability and reliability in more complex and varied real-world scenarios.

## Figures and Tables

**Table 1:** Execution time of each sub-task and the full task, mean and standard deviation over 10 executions.

Sub-Task	Time (s)
Obtain Positions	1.4±0.2
Pick Bottle	20.6±0.8
Grasp Cap	22.1±0.5
Unscrew	16.3±1.4
Total	60.4±2.9

**Table 2:** Execution time of unscrew process with different methods: force-torque control, slippage sensor, human demonstration and our approach, force control with DRL.

Method	Time (s)
Force-torque control	100
Slippage sensors	20
Human demonstration	30
Force control and DRL	16

## Data Availability

The original contributions presented in the study are included in the article, further inquiries can be directed to the corresponding author.

## Abbreviations

The following abbreviations are used in this manuscript:

AR	Assistive Robotics
DRL	Deep Reinforcement Learning
FK	Forward Kinematics
IK	Inverse Kinematics
ISBT	International Society of Beverage Technology
MADRL	Multi Agent Deep Reinforcement Learning MADRL
MDP	Markov Decision Process
RL	Reinforcement Learning
ROS	Robot Operating System
SBL	Single-query Bi-directional Lazy collision checking
